# Marginal Contribution-Based Distributed Subchannel Allocation in Small Cell Networks

**DOI:** 10.3390/s18051500

**Published:** 2018-05-10

**Authors:** Shashi Shah, Somsak Kittipiyakul, Yuto Lim, Yasuo Tan

**Affiliations:** 1 School of Information, Computer, and Communication Technology, Sirindhorn International Institute of Technology (SIIT), Thammasat University (TU), Pathum Thani 12121, Thailand; somsak@siit.tu.ac.th; 2 School of Information Science, Japan Advanced Institute of Science and Technology (JAIST), Ishikawa 923-1292, Japan; ylim@jaist.ac.jp (Y.L.); ytan@jaist.ac.jp (Y.T.)

**Keywords:** small cells, distributed subchannel allocation, game theory, best-response, potential games, marginal contribution

## Abstract

The paper presents a game theoretic solution for distributed subchannel allocation problem in small cell networks (SCNs) analyzed under the physical interference model. The objective is to find a distributed solution that maximizes the welfare of the SCNs, defined as the total system capacity. Although the problem can be addressed through best-response (BR) dynamics, the existence of a steady-state solution, i.e., a pure strategy Nash equilibrium (NE), cannot be guaranteed. Potential games (PGs) ensure convergence to a pure strategy NE when players rationally play according to some specified learning rules. However, such a performance guarantee comes at the expense of complete knowledge of the SCNs. To overcome such requirements, properties of PGs are exploited for scalable implementations, where we utilize the concept of marginal contribution (MC) as a tool to design learning rules of players’ utility and propose the marginal contribution-based best-response (MCBR) algorithm of low computational complexity for the distributed subchannel allocation problem. Finally, we validate and evaluate the proposed scheme through simulations for various performance metrics.

## 1. Introduction

Small cells, which are typically characterized as providing dynamic range coverage and low-powered radio access nodes overlaid on macrocells, provide more flexible and scalable deployment by reusing available radio resources [1]. Deployment of small cells decreases the load of macrocell base stations (MBSs), provides a better link with a reduced distance between serving base stations (BSs) and mobile stations (MSs), and enhances network performance with improved capacity. However, the advantages of small cells could be insufficient in dense and random networks due to strong co-channel interference from neighboring small cells utilizing common radio resources, such as subchannels in orthogonal frequency division multiple access (OFDMA) networks [2]. Resource allocation becomes a critical issue and a challenge as it induces interference in the network, restricting both capacity and cellular coverage [3].

Centralized approaches for resource allocation [4,5,6,7] require some central authority to maintain complete knowledge of the environment through frequent information exchange with small cell base stations (SBSs). However, such requirements may not be desirable when considering randomly deployed large numbers of small cells, due to the computational complexity of the centralized solution [8]. As the number and location of small cells become unpredictable, mobile operators should consider distributed approaches allowing SBSs to self-organize by learning their dynamic environment, and gradually allocate resources.

Game theory [9], as a distributed approach for resource allocation, is suitable for modeling the individualistic behaviors of small cells, where interactions among multiple decision-makers (players) over common resources can be formulated and analyzed as a game. Recently, researchers have proposed distributed learning rules for the problem of resource allocation [10,11,12,13,14,15,16], which can be generalized in two ways: (1) as uncoordinated; and (2) as coordinated. Some uncoordinated learning rules, such as spatial adaptive play [10], utility-based learning [11], stochastic-learning-automata [12], and reinforcement learning [13], are completely uncoupled, i.e., the resource allocation procedure does not depend on the actions of anyone else. With restriction to information exchange, the achieved solutions of these approaches have a trade-off in terms of convergence speed and efficiency. Coordinated learning rules, for example, cloud-assisted best-response [14] and cluster formation [15,16], require some coordination mechanism such that a player is selected to opportunistically update its strategy at a given time. Although it maintains fairness by providing equal opportunity to the decision-makers, such a greedy adaption-based resource allocation, in general, does not necessarily result in a steady-state solution. Nevertheless, the distributed approaches have the advantages of scalable implementation, but their performance is typically inferior compared to the centralized approaches. In this regard, while distributed decision-makers are opportunistically trying to maximize their own performance, it is necessary to implement some mechanisms in their learning rules such that stable resource allocation can be obtained, while efficiency and fairness are also maintained within the network.

In this paper, we focus on coordinated learning rules for resource allocation and model OFDMA-based small cell networks (SCNs) consisting of SBSs as distributed decision-makers trying to select a proper subchannel for transmission. To guarantee for existence of a steady-state solution, we resort to a class of potential game (PG) called the exact potential game (EPG) [17], and utilize the concept of marginal contribution (MC) [8] as a mechanism to design the welfare-based learning rule to achieve efficient and fair resource allocations. However, the performance guarantee of EPGs comes at the expense of complete information of the environment [8], i.e., decision-makers usually require overall information from other remaining decision-makers in the network [18,19], making the solution unscalable. To overcome the requirement of global information, we propose the marginal contribution based best-response (MCBR) algorithm of low computational complexity by modeling information exchange requirements only on a specific subchannel of the neighborhood. The main contributions of this paper are summarized as below:

- For the objective of finding distributed subchannel allocation in SCNs, the concept of MC is utilized to design a welfare-based learning rule for the decision-makers. It is shown that efficient and fair subchannel allocation is achievable in a distributed network if some sort of self-awareness is introduced to decision-makers’ learning rules, such that each decision-maker will adapt to improve some measure of the influence caused by its actions to others in the network in addition to improving its own performance.
- The welfare-based learning rule maintains the universal properties of an EPG; hence convergence to a pure strategy NE is predictive and guaranteed through best-response (BR) dynamics. Moreover, the proposed MCBR algorithm overcomes the short-comings of EPG for practical implementations in the distributed networks, i.e., complete information of the environment. Here, a decision-maker utilizes the concept of probability distribution in order to select a new subchannel for sensing, and requires information feedback only from those neighboring decision-makers currently using the subchannel.

The wireless cellular networks have witnessed an exponential growth in mobile data traffic, mainly due to the popularity of multimedia services and high data rate applications [20]. One of the key emerging trend to accommodate such demand is through network densification, such as extensive deployment of SBSs, by reusing radio resources [21]. Network densification with small cells, which results in spatial densification, has attracted much attention [22,23,24] due to its promising network performance with improvement in capacity. Several studies have provided prospects on it from the perspective of key challenges [3,24,25], identifying interference management and resource allocation as the most crucial issues that can limit the gains anticipated. The centralized approaches [4,5,6,7] for solving the resource allocation problem in random and dense SCNs are not scalable due to the requirement of global information to manage the le network. On the contrary, convergence speeds of the distributed approaches that require no information exchange [10,11,12,13] are very slow, giving rise to the inherent limitations for practical implementation in large-scale networks with extensive deployment of SBSs. In this work, unlike the traditional opportunistic learning rules in most of the previous studies [14,15,16] where each decision-maker acts rationally to maximize their individual performance, we show that implementing the proposed mechanism of the welfare-based learning rule for decision-makers provides network-wide performance improvement with a fair and stable resource allocation solution. Moreover, it is suitable for practical implementation as it requires only local and limited information exchange, which results in low and scalable computational complexity.

Recently, the concept of network sharing has also been investigated to allow operators to share their infrastructures in order to maximize the use of existing radio resources while simultaneously minimizing the operational costs [26,27,28,29,30]. Our work facilitates these recent trends and can be applied as a tool to achieve the much anticipated gains through deployment of small cells by efficient allocation of the radio resources in a distributed fashion. If is of note that the proposed distributed subchannel allocation scheme can also be applied to wireless sensor networks (WSNs) to assist resource-constrained sensors in realizing efficient utilization of the spectrum.

The rest of this paper is organized as follows. In Section 2, we define the system model for the SCNs. The game theoretic framework and the proposed learning rule for decision-makers are introduced in Section 3. The proposed distributed subchannel allocation algorithm is briefly described in Section 4. Section 5 presents the simulation results for different scenarios to demonstrate the performance gains with our proposed scheme. Finally, Section 6 summarizes this paper.

## 2. System Model

**Figure 1:**
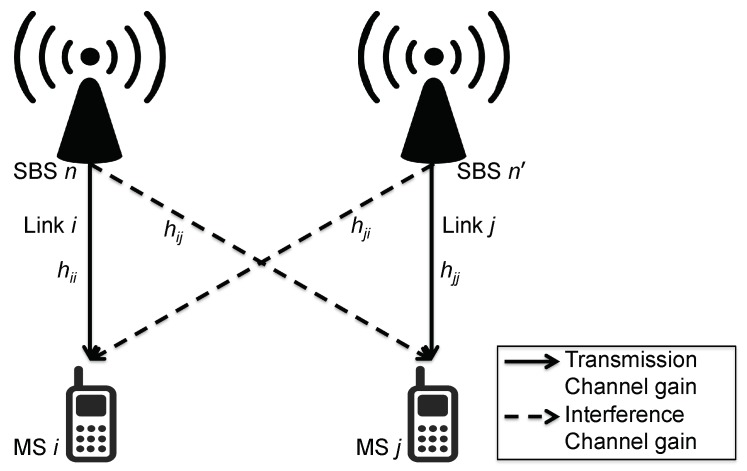
System model. SBS: small cell base station; MS: mobile station.

We consider a downlink subchannel allocation in an OFDMA-based SCN, as shown in Figure 1, that consists of *N* transmitting SBSs and *M* receiving MSs where N≤M. Let N={1,…,N} be the set of SBSs, and M={1,…,M} be the set of MSs. Each SBS takes care of a non-overlapping set of MSs. Then, ∀n∈N, $M={⋃}_{n=1}^{N}M_{n}$, and $M_{n}=\left|M_{n}\right|$ is the number of MSs associated with SBS *n*. Here, a link i∈M is between one transmitting SBS and one receiving MS. Hence, the total number of links is equal to *M*. The spectrum bandwidth *B* of the OFDMA network is divided into *K* subchannels. The set of subchannels is denoted as K={1,…,K}, with each subchannel having a bandwidth of B/K.

Consider $h_{ii}\left(k\right)$ to be the channel gain of transmission on link *i*, i.e., the channel gain from the transmitting SBS of link *i* to the receiving MS of link *i*, on subchannel k∈K. Hence, $h_{ji}\left(k\right)$ represents the channel gain of interference from the transmitting SBS of link *j* to the receiving MS of link *i* on subchannel *k*. The transmit power of link *i* which is using subchannel *k* is $P_{i,k}$. We assume all links are active simultaneously and each link utilizes only one subchannel. Hence, if the number of links transmitted by SBS *n* is $M_{n}$, then $P_{i,k}=P_{\max}/M_{n}$, where $P_{\max}$ is the maximum transmit power of each SBS. If the link *i* does not use subchannel $k^{\prime }\in K$ then $P_{i,k^{\prime }}=0$.

In this work, we use the physical interference model which takes into account interference produced by all other links using the same subchannel. Hence, the downlink signal-to-interference-plus-noise ratio (SINR) for link *i* on subchannel *k* is given by

(1) $$\gamma_{i,k}=\frac{P_{i,k}h_{ii}\left(k\right)}{I_{i,k}+\sigma^{2}}$$

where $I_{i,k}$ is the downlink co-channel interference to link *i* on subchannel *k*, i.e., $I_{i,k}$ is the sum of the co-channel interference from all other links using the same subchannel *k*, $I_{i,k}=\sum_{j\in M,j\neq i}P_{j,k}h_{ji}\left(k\right),$ and $\sigma^{2}$ is the additive white Gaussian noise (AWGN) power. From Shannon theory, the capacity of link *i* using subchannel *k* is

(2) $$C_{i}=\frac{B}{K}log_{2}\left(1+\gamma_{i,k}\right)$$

The total capacity of the network is $C=\sum_{i=1}^{M}C_{i}$.

The notations used in this paper are summarized in Table 1.

## 3. Game Theoretic Framework and Utility Function Design

In this section, we describe the game theoretic framework to model the interaction between different SBSs to decide which subchannel to use for each link. We describe the BR strategy and the Nash equilibrium (NE) solution. Although it is well known that the BR strategy under sequential updates can lead to a pure strategy NE, here we show by an example that this is not the case for our problem. Hence, we direct our attention to PGs, which under sequential updates can lead to a pure strategy NE. We consider a set of *M* number of players, i.e. the total number of links in the SCN, where all other players except a given player *i* are denoted by “−i”. The set of strategies of player *i* is a set of all available subchannels, denoted by $S_{i}=\left\{1,2,\ldots ,K\right\}$. Now, the Cartesian product $S_{M}\;=\;\prod_{j\in M}S_{j}$ consists of set of all ordered pairs of strategy profiles. For a strategy $s_{j}\in S_{j}$, an ordered pair $s\;=\;{\left(s_{j}\right)}_{j\;\in \;M}\;\in \;S_{M}$, such that $s=\left(s_{i},s_{-i}\right)=\left(s_{1},\ldots ,s_{M}\right)\in S_{M}$, and $s_{-i}=\left(s_{1},\ldots ,s_{i-1},s_{i+1},\ldots ,s_{M}\right)\in S_{M\setminus \{i\}}$. For simplicity, we use S and $S_{-i}$ to denote $S_{M}$ and $S_{M\setminus \{i\}}$ respectively. The utility function of player *i*, $u_{i}:S_{i}\to R$, maps the strategy profile to a real value R. Now given opponents’ strategies, player *i* selects a strategy among all available strategies such that its own utility is maximized, i.e., player *i* prefers strategy $s_{i}$ to $s_{i}^{\prime }$ if $u_{i}\left(s_{i},s_{-i}\right)\geq u_{i}\left(s_{i}^{\prime },s_{-i}\right)$. Such a strategy is called BR strategy [31]. The BR strategy of player *i* to the profile of strategies $s_{-i}$ played by all other opponents, $BR_{i}\left(s_{-i}\right)$, is a strategy $s_{i}$ such that,

(3) $$BR_{i}\left(s_{-i}\right)=\arg\max_{s_{i}\in S_{i}}u_{i}\left(s_{i},s_{-i}\right)$$

where $u_{i}$ is the utility of player *i* and $S_{i}$ is the set of all possible strategies of player *i*. Hence, following Equation (3), player *i* best-responds to the most recently chosen strategies of the other opponents and selects a strategy, $s_{i}$, from the set of all possible strategies, $S_{i}$, that maximizes its utility function.

The commonly used solution concept in game theory is NE [9], which indicates a steady-state condition for strategies of all players. At NE, no player has an incentive to unilaterally deviate from the steady state. A strategy profile $s^{*}=\left(s_{1}^{*},\ldots ,s_{M}^{*}\right)$ constitutes an NE if it satisfies the following condition

(4) $$u_{i}\left(s_{i}^{*},s_{-i}^{*}\right)\geq u_{i}\left(s_{i},s_{-i}^{*}\right),\forall s_{i}\neq s_{i}^{*},\forall s_{i}\in S_{i},\forall i\in M$$

Although NE is a solution concept for many games when players update their strategy sequentially under BR dynamics, the existence of a pure strategy NE is not always guaranteed for our problem of distributed subchannel allocation, as shown in Example 1.

**Example** **1.** *Consider a subchannel allocation game in the SBS and MS pairs arrangement shown in Figure 1. Let N={1,2,3}, K={1,2}, M={1,2,3} and $M_{n}=1$ for all n∈N. Let $h_{11},h_{22}$, and $h_{33}$ be the channel gains for transmission, and the channel gains of interference are given by $h_{21}>h_{31},h_{32}>h_{12}$, and $h_{13}>h_{23}$, respectively. Let Equation (1) be the utility function to be maximized by link i∈M at time slot t, such that*

(5) $$u_{i}\left(s_{i}\left(t\right)=k\right)=\gamma_{i,k}$$

*The utility function in Equation (5) corresponds to utility obtained by link i on subchannel k assuming $s_{-i}\left(t\right)=s_{-i}\left(t-1\right)$, i.e., all other links continue to use the subchannel selected in a previous time slot. This subchannel allocation game has eight possible subchannel allocation profiles, $\left(s_{1},s_{2},s_{3}\right)$; two same-subchannel profiles are (1,1,1) and (2,2,2), and six mixed-subchannel profiles are (1,1,2),(1,2,1),(1,2,2),(2,1,1),(2,1,2) and (2,2,1).*

*Case I: If initially at time slot t=0, a same-subchannel profile is considered, say $\left(s_{1},s_{2},s_{3}\right)=\left(1,1,1\right)$, then all links have an incentive to change subchannel at next time slot t=1 since $BR_{i}\left(s_{-i}\right)=\left\{2\right\}$, ∀i∈M. This will change the strategy profile (1,1,1) to any one of the mixed-subchannel profiles: (1,1,2),(1,2,1) or (2,1,1), depending on which link is chosen to update their strategy at the next time slot t=1.*

*Case II: If initially at time slot t=0 a mixed-subchannel profile is considered, say $\left(s_{1},s_{2},s_{3}\right)=\left(1,1,2\right)$, then at next time slot t=1, link 1 has an incentive to change to subchannel 2 since $BR_{1}\left(s_{-i}\right)=\left\{2\right\}$. The sequence of subchannel switching is (1,1,2)→(2,1,2)→(2,1,1)→(2,2,1)→(1,2,1)→(1,2,2)→(1,1,2)→…; i.e., it does not converge to a pure strategy NE. In fact, none of the six mixed-subchannel profiles can satisfy Equation (4). This subchannel allocation game does not converge to a pure strategy NE.*

### 3.1. Potential Games and Their Characteristics

To guarantee that the subchannel allocation game converges to a pure strategy NE, we focus on a class of strategic form games called PG [17], which ensures convergence to a pure strategy NE through BR dynamics. Specifically, a class of PG called EPG has at least one pure strategy NE [17], and is characterized by the existence of a potential function, P:S→R, with property,

(6) $$u_{i}\left(s_{i},s_{-i}\right)-u_{i}\left(s_{i^{\prime }},s_{-i}\right)=P\left(s_{i},s_{-i}\right)-P\left(s_{i^{\prime }},s_{-i}\right),$$

for all $i\in M,s_{i},s_{i^{\prime }}\in S_{i}$, and $s_{-i}\in S_{-i}$.

Here, the potential function acts as a global performance indicator, such that any increments/decrements in player’s utility from the change in strategy are equal to the same increments/decrements in the overall networks’ utility. In other words, at any instance of play, a individual player’s best strategy is also the best strategy for the network. As such, the maxima of the potential function corresponds to a pure strategy NE [17]. Hence, with sequential updates of strategy by players following BR dynamics, the process will converge to a pure strategy NE regardless of the order of play and the initial strategy profiles of the game.

In order to capture benefits associated with an EPG, it is desirable to design the utility function for players according to some specified learning rules. This can provide predictive performance in the subchannel allocation game when players rationally play according to BR dynamics. Desirably, to optimize the total capacity of the SCN while players distributively chooses their strategies, a simple and straightforward option would be to assign the players’ utility function and potential function equal to the total capacity (similar to the identical interest games [32,33]), such that:

(7) $$u_{i}\left(s_{i},s_{-i}\right)=P\left(S\right)=C$$

However, for the utility function in Equation (7), each link *i* would require information feedback from all other links, which would become extremely complicated in a dense SCN. Looking forward to reduce the computational complexities while keeping the objective to maximize total capacity of the SCN, we resort to utilizing design rule of utility function based on the concept of MC that is also proven to be an EPG [8]. The concept of MC considers designing utility function derived directly from the potential function, and is defined as [8],

(8) $$\begin{array}{c} u_{i}^{\mathrm{MC}}\left(s\right)=P^{\mathrm{MC}}\left(s\right)-P^{\mathrm{MC}}\left(s_{-i}\right) \end{array}$$

where $P^{\mathrm{MC}}\left(s\right)$ and $P^{\mathrm{MC}}\left(s_{-i}\right)$ are the value of potential function with presence and absence of player *i* in the game, respectively.

### 3.2. Utility Function Design with Limited Information Feedback

We consider a potential function, $P^{\mathrm{MC}}:S\to R$, with the objective to maximize total capacity of the SCN for transmission of link i∈M on subchannel *k*, such that,

(9) $$\begin{array}{cc} P^{\mathrm{MC}}\left(S\right) & =\sum_{k}W_{k}\left(s\right) \end{array}$$

where $W_{k}\left(s\right)=\sum_{i:s_{i}=k}C_{i,k}$ is the total welfare (in term of capacity) from all links that are using subchannel *k*. Hence, the potential function $P^{\mathrm{MC}}\left(S\right)$ essentially reflects overall capacity in Equation (2).

From Equations (8) and (9), we can express the utility of link *i* when it is using subchannel $k^{\prime }$, i.e., $s_{i}=k^{\prime }$, while the other links are using strategies $s_{-i}$ as,

(10) $$\begin{array}{cc} u_{i}^{\mathrm{MC}}\left(s_{i}=k^{\prime },s_{-i}\right) & =P^{\mathrm{MC}}\left(s_{i}=k^{\prime },s_{-i}\right)-P^{\mathrm{MC}}\left(s_{-i}\right)\\ & =\sum_{k}W_{k}\left(s\right)-\sum_{k}W_{k}\left(s_{-i}\right)\\ & =(W_{k^{\prime }}\left(s\right)+\sum_{k\neq k^{\prime }}W_{k}\left(s_{-i}\right))-(W_{k^{\prime }}\left(s_{-i}\right)+\sum_{k\neq k^{\prime }}W_{k}\left(s_{-i}\right))\\ & =W_{k^{\prime }}\left(s_{i},s_{-i}\right)-W_{k^{\prime }}\left(s_{-i}\right) \end{array}$$

where $W_{k^{\prime }}\left(s_{i},s_{-i}\right)$ and $W_{k^{\prime }}\left(s_{-i}\right)$ are the total welfare on subchannel $k^{\prime }$ with presence and absence of link *i* in the game, respectively. Here while learning the utility on subchannel $k^{\prime }$ to improve its own performance, the link *i* is aware of its influence to other links that are using subchannel $k^{\prime }$. From Equation (10), since link *i* is using only subchannel $k^{\prime }$, the total welfare on all other subchannels, $k\neq k^{\prime }$, is the same as that without link *i*, i.e., we have for any subchannel $k\neq k^{\prime }$, $W_{k}\left(s_{i}=k^{\prime },s_{-i}\right)=W_{k}\left(s_{-i}\right)$ and hence the total welfare from all other subchannels (except $k^{\prime }$) is the same with and without link *i*. With this observation, the utility of link *i* is expressed simply as $u_{i}\left(s_{i}=k^{\prime },s_{-i}\right)=W_{k^{\prime }}\left(s_{i},s_{-i}\right)-W_{k^{\prime }}\left(s_{-i}\right)$, which is the additional welfare on subchannel $k^{\prime }$ when link *i* is in the game, compared to that when link *i* is not in the game, assuming all other links stay using the same strategies $s_{-i}$. Hence, the computational complexities to compute the utility are reduced in this game, since link *i* would require information feedback only from those links that are also using subchannel $k^{\prime }$, i.e., for which the strategy is $s_{-i}=k^{\prime }$.

The game with utility function in Equation (10) is an EPG for the potential function in Equation (9) as shown in Theorem 1.

**Theorem** **1.** *The game $G=(M,\left\{S\right\},\left\{u_{i}^{\mathrm{MC}}\left(s\right)\right\})$ is an EPG and has at least one pure strategy NE.*

**Proof** **of** **Theorem** **1.** As shown in Section 3.2.2. of [8], when we set MC as a utility function, the game with this utility function is proven to be an EPG where the strategy profile that maximizes the potential function is the pure strategy NE. ☐

**Corollary** **1.** *For a particular initial subchannel profile and sequence of play, the pure strategy NE of the game $G=(M,\left\{S\right\},\left\{u_{i}^{\mathrm{MC}}\left(s\right)\right\})$ is unique.*

**Proof**  **of** **Corollary** **1.** Consider an initial subchannel profile, $\left(s_{1},\ldots ,s_{i},\ldots ,s_{M}\right)$ where ∀i∈M, and there is a strategy update sequence of players, say ${\left(m\right)}_{m\in M}$.

Since the game $G=(M,\left\{S\right\},\left\{u_{i}^{\mathrm{MC}}\left(s\right)\right\})$ is an EPG, the potential function $P^{\mathrm{MC}}\left(S\right)$ is guaranteed to converge to a maxima and that maxima in turn corresponds to a pure strategy NE [17]. Hence, a pure strategy NE is unique, depending on the initial subchannel profile and the sequence of play for the strategy update by players. ☐

### 3.3. Information Feedback from Neighboring Small Cells

Note that, to compute the utility on a subchannel in Equation (10), the serving SBS of a link needs to gather information of all other links in the SCN that are utilizing the particular subchannel. Here, it is assumed that the transmission on a link interferes with all other links over the common subchannel. However, this is not always true in SCNs where the interference sources are dominant only within a neighborhood due to low transmitting power of SBSs [25]. In this regard, we consider each MS aperiodically listens the network to construct a neighbor relation table (NRT) that contains the identity of its neighboring SBSs, and sends back to its serving SBS. For this purpose, the automatic neighbor relation framework introduced in the third-generation partnership project (3GPP) can be utilized to discover neighboring SBSs of the MS [34]. Here, each SBS broadcasts a unique physical cell identity (PCI), the identifier of the SBS, which the MSs can detect if the reference signal receive power (RSRP) is greater than some threshold value ($RSRP_{TH}$). For simplicity of evaluation, we assume that the RSRP from a SBS is greater than the $RSRP_{TH}$ within their coverage range. Now, the MS lists all the detected PCIs in its NRT, indicating them as its neighboring SBSs, and sends the NRT to the serving SBS. With the PCIs information in the NRT, the serving SBS can establish X2 interface connections with the neighboring SBSs of the MS in order to receive necessary information feedback while computing the utility (the X2 interface allows for inter-cell signaling between SBSs) [35]. Hence, to compute the utility on a subchannel, the serving SBS of a link needs information feedback only of those links originating from the neighboring SBSs that are utilizing the particular subchannel.

Now, the utility function in Equation (10) of link *i* is modified as

(11) $$\begin{array}{c} u_{i}^{\mathrm{MC}}\left(s_{i}=k^{\prime },s_{j}\right)=W_{k^{\prime }}\left(s_{i},s_{j}\right)-W_{k^{\prime }}\left(s_{j}\right) \end{array}$$

Here, $j\in J_{i}\subset M$ is the set of links originating from the neighboring SBSs of link *i*. The utility function in Equation (11) for link *i* is calculated with the feedback of information only of the links in set $J_{i}$ that are also using subchannel $k^{\prime }$, i.e., for which the strategy is $s_{j}=k^{\prime }$, thus further reducing the computational complexity to compute the utilities.

## 4. Distributed Algorithm for Subchannel Allocation

In this section, we present a distributed algorithm used by each SBS for subchannel allocation of all links that it handles. In general, there may be a large number of links in a SCN, and the amount of subchannel sensing and information exchange can be overwhelming. Hence, we are looking to limit the amount of subchannel sensing and information exchange requirements. For subchannel allocation, the proposed MCBR algorithm makes decisions based on information obtained from sensing the subchannels where the number of new sensing subchannel is limited to one. Moreover, for the particular sensing subchannel, the algorithm requires information only from those other links that are currently allocated to that subchannel.

### 4.1. Marginal Contribution-Based Best-Response Algorithm

MCBR is described in Algorithm 1. Here, we assume that all SBSs are synchronized and time is divided into time slots of a fixed duration. Each time slot is further divided into two phases, called the *sensing* phase and the *payload* phase. Here, we consider the sensing phase alternating with the payload phase. During the sensing phase, SBSs learn the utilities for different subchannels and make the decision to select the subchannel providing with highest utility. In the payload phase, the SBS transmits on the selected subchannel.

In the sensing phase of time slot *t*, a link *i* is selected to update its strategy. During this sensing phase, all other links keep transmitting on the subchannel used in the previous time slot t−1. The link *i* selects a new subchannel for sensing according to the subchannel selection probability vector $p_{i}$. Here, $p_{i}=\left\{p_{i,1}\left(t\right),\ldots ,p_{i,K}\left(t\right)\right\}$, where each element $p_{i,k}\left(t\right)$ is the probability of choosing subchannel *k* at time slot *t* by link *i*. The SBS for link *i* measures the utilities on the new sensing subchannel and the subchannel allocated in the previous time slot, i.e., for strategies $s_{i}=k^{\prime }$ and $s_{i}=k_{t-1}$, according to Equation (11). Note that, in order to calculate the two utilities, link *i* only needs to receive capacity reports of links $j\in J_{i}$ that are transmitting on those two subchannels.

Now, the subchannel with highest utility is determined based on the BR dynamics, such that,

(12) $$BR_{i}\left(s_{j}\right)=\arg\max_{s_{i}\in \left\{k^{\prime },k_{t-1}\right\}}u_{i}^{\mathrm{MC}}\left(s_{i},s_{j}\right)$$

From Equation (12), link *i* would best respond to switch to the new sensing subchannel $k^{\prime }$ if the utility $u_{i}^{\mathrm{MC}}\left(s_{i}=k^{\prime },s_{j}\right)>u_{i}^{\mathrm{MC}}\left(s_{i},=k_{t-1},s_{j}\right)$. Otherwise, link *i* would stay with the subchannel selected in the previous time slot, i.e., subchannel $k_{t-1}$.

**Algorithm 1** MCBR used by SBSs for links it handles.
01: Initialization: (at time slot t=0)

02:   Get synchronization

03:   Set subchannel selection probability of each link as
$$p_{i,k}\left(t\right)=\frac{1}{K},\;\forall i\in M,k\in K$$

04:   Randomly select a subchannel $k_{t}$ for all links *i* according to the subchannel selection probability vector $p_{i}$

05: In **sensing phase** of time slot t=1,2,…:

06:   Select a link *i* in predetermined round-robin fashion

07:   Select a new sensing subchannel $k^{\prime }$ according to $p_{i}$

08:   Receive capacity reports of links $j\in J_{i}$ for strategies $s_{i}=k_{t-1}$ and $s_{i}=k^{\prime }$

09:   Measure utilities according to Equation (11) for strategies $s_{i}=k_{t-1}$ and $s_{i}=k^{\prime }$

10:   Determine a subchannel $k_{t}$ with the highest utility according to Equation (12)

11:   Update subchannel selection probabilities such that,
$$p_{i,k}\left(t\right)=\left\{\begin{array}{c} p_{i,k}\left(t-1\right)+\beta {\tilde{r}}_{i}\left(1-p_{i,k}\left(t-1\right)\right),\;\mathrm{if}\;k=k_{t}\\ p_{i,k}\left(t-1\right)-\beta {\tilde{r}}_{i}p_{i,k}\left(t-1\right),\;\mathrm{otherwise} \end{array}\right.$$

    where $0<\beta =\frac{1}{K}<1$ is learning step-size, and ${\tilde{r}}_{i}$ is normalized utility increment given by
$${\tilde{r}}_{i}=\frac{u_{i}^{\mathrm{MC}}\left(s_{i}=k_{t},s_{j}\right)}{u_{i}^{\mathrm{MC}}\left(s_{i}=k_{t},s_{j}\right)+u_{i}^{\mathrm{MC}}\left(s_{i}=k_{t-1},s_{j}\right)}$$

12: In **payload phase** of time slot *t*:

13:   Link *i* transmits on subchannel $k_{t}$

14:   Set t←t+1, go to step 05


At the end of the sensing phase, the SBS for link *i* chooses the subchannel with the highest utility for transmission in the payload phase (the rest of time slot *t*) and updates the subchannel selection probabilities in $p_{i}$ according to the received utility as shown in step 11. The updates of subchannel selection probabilities are dependent on learning the step size (β), normalized utility increment (${\tilde{r}}_{i}$), and the selected subchannel ($k_{t}$) at time slot *t*. To understand the update process, let us again consider example 1 with two subchannels K={1,2}. Suppose for link *i* at time slot *t* the subchannel selection probability vector is $p_{i}=\left\{p_{i,1}\left(t-1\right),p_{i,2}\left(t-1\right)\right\}=\left\{0.5,0.5\right\}$ with $\beta =0.5,{\tilde{r}}_{i}=0.5$, and subchannel 1 is selected for transmission in the payload phase, i.e., $k_{t}=1$. From the update rule in step 11, since link *i* has selected subchannel 1, the probability of selecting this subchannel in the next time slot increases and is given by $p_{i,1}\left(t\right)=0.5+\left(0.5\right).\left(0.5\right).\left(1-0.5\right)=0.625$. On the other hand, the probability of selecting subchannel 2 in the next time slot decreases and is given by $p_{i,2}\left(t\right)=0.5-\left(0.5\right).\left(0.5\right).\left(0.5\right)=0.375$. Note that the sum of subchannel selection probabilities of link *i* at time slot *t* always equals to 1, i.e., $\sum_{k\in K}p_{i,k}\left(t\right)=1$. Such an approach fpr selecting a new sensing subchannel based on the concept of probability distribution allows for the exploration of more subchannel profiles while minimizing the amount of subchannel sensing and information exchange requirements at each time slot.

### 4.2. Computational Complexity Analysis

At each iteration in MCBR algorithm, the sensing phase complexity is O(2T) which results while sensing the new subchannel and the subchannel allocated in the previous time slot, where *T* is the duration of the sensing time. The complexity for information feedback from the neighboring links is reflected by $O(|J_{i}|)$, the upper bound, and Ω(0), the lower bound. This range corresponds to the two extreme cases when the sensing subchannel is also allocated by all other neighboring links and none, respectively. Certainly, the overall computational complexity depends on the number of iterations needed for steady-state convergence, which is not unlimited because of a finite number of possible strategy profiles. In contrast, the identical interest game, which is shown to be an EPG [32,33], with a potential function and utility function equal to Equation (7), has the sensing phase complexity of O(|K|T) and the information feedback complexity of O(|M|) at each iteration. This is not scalable and would result in significant computational complexity in dense SCN scenarios with large numbers of subchannels. Therefore, the computational complexity of the MCBR algorithm for each iteration is comparatively low, scalable, and suitable for practical implementation.

## 5. Simulation Results

In this section, we simulate and compare performance of the proposed MCBR algorithm with information feedback from the neighborhood, denoted as MCBR (Neighborhood Information), for different performance metrics. The simulations are performed in MATLAB version R2017b 9.3.0.713579.

We consider deployment of SBSs that belong to several operators in a cluster [36]. The small cell cluster consists of SBSs uniformly deployed in a circular area of radius *R* meters. MSs are uniformly distributed around circular coverage of radius $r_{n}$ meters of SBSs. For simulations, we choose R=100 m and $r_{n}=30$ m. The propagation model considered is the 3GPP Model 1 (Table A.2.1.1.2-3) in [37]. The channel gains include path-loss, lognormal shadowing, and multipath Rayleigh fading. The lists of parameters used for simulation are shown in Table 2.

We evaluate performance of the proposed algorithm in three different radio resource scenarios, namely: sparse, moderate, and dense scenarios, where the numbers of SBSs and MSs, (N,M), are set to (10,15),(10,55), and (10,100), respectively. The reason for considering the three radio resource scenarios is to show the effectiveness of our proposed scheme in different types of scenarios. For the sparse scenario, the evaluated performance metrics include effects of varying the number of subchannels, convergence analysis, and efficiency comparison with other schemes. One reason for considering the sparse scenario to show the efficiency comparison is that the search for global optimum using an exhaustive search can be performed, while it is intolerably time consuming in the other two scenarios. Finally, for all the three scenarios, we compare the performance of MCBR (Neighborhood Information) with other schemes in terms of Jain’s fairness index, average system capacity, average interference, and information feedback requirements from an average number of links.

For comparison, we consider the BR strategy with utility in Equation (2) for two different schemes, namely, BR (Simultaneous) and BR (Sequential) [31]. In the simultaneous scheme, all links update their strategies simultaneously, without knowing the strategies that have been chosen by other links. Hence, they are making a simultaneous move. In the sequential scheme, each link take turns to update their strategies for selecting a subchannel that maximizes their utility. We also compare the performance of the MCBR algorithm for the utility function described in Equation (10), i.e, with complete information feedback from all other links using a particular subchannel in the SCN, denoted as MCBR (Complete Information).

### 5.1. Performance in Sparse Scenario

In this subsection, we compare the performance of MCBR (Neighborhood Information) in the sparse scenario.

#### 5.1.1. Effects of Varying the Number of Subchannels

**Figure 2:**
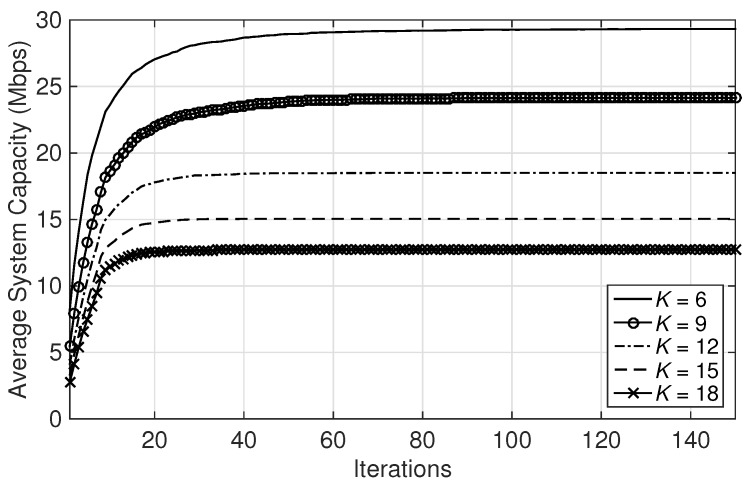
Average system capacity with varying numbers of subchannels.

First of all, the effect of varying the number of subchannels in MCBR (Neighborhood Information) is demonstrated for K={6,9,12,15,18}. In Figure 2, it is noted that as the number of subchannel increases, the subchannel allocation collision decreases; hence the convergence speed to a steady state increases. However, since the total bandwidth is divided with the number of subchannels in Equation (2), the achieved total capacity decreases with an increase in subchannels.

For simplicity, in the rest of simulations, we fix the number of subchannels to six.

#### 5.1.2. Convergence Analysis

**Figure 3:**
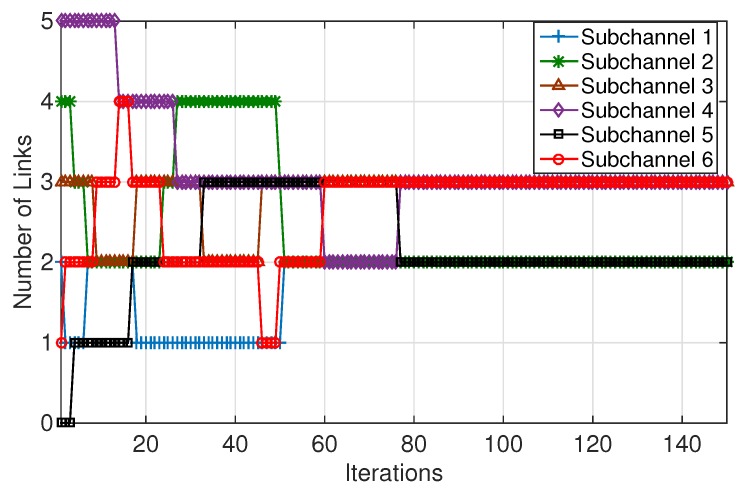
The evolution of number of links on each of the subchannels.

To show the convergence of MCBR (Neighborhood Information), Figure 3 depicts the evolution of the number of links on each subchannel. It is seen that the subchannel allocation converges to a steady state in about 78 iterations. This observation validates the convergence of the proposed MCBR (Neighborhood Information) algorithm.

#### 5.1.3. Efficiency Comparison

**Figure 4:**
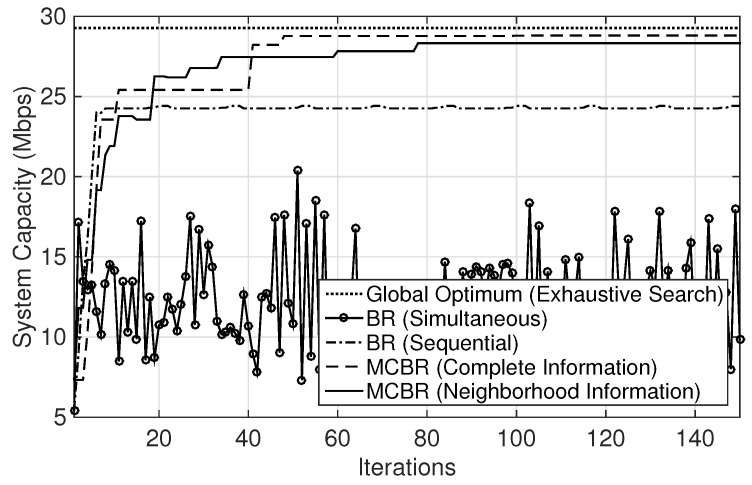
System capacity comparison. MCBR: marginal contribution-based best-response.

Figure 4 tracks the change in system capacity at each iteration and also shows the efficiency of the steady-state convergence. It is noted that MCBR (Neighborhood Information) and MCBR (Complete Information) converge to steady states after some iterations, while BR (Sequential) and BR (Simultaneous) do not achieve any steady-state solution. Also, the plots for both MCBR (Neighborhood Information) and MCBR (Complete Information) exhibit the convergence to the pure strategy NE, corresponding to maxima at about 78 and 49 iterations, respectively. This validates that MCBR algorithm is an EPG.

**Figure 5:**
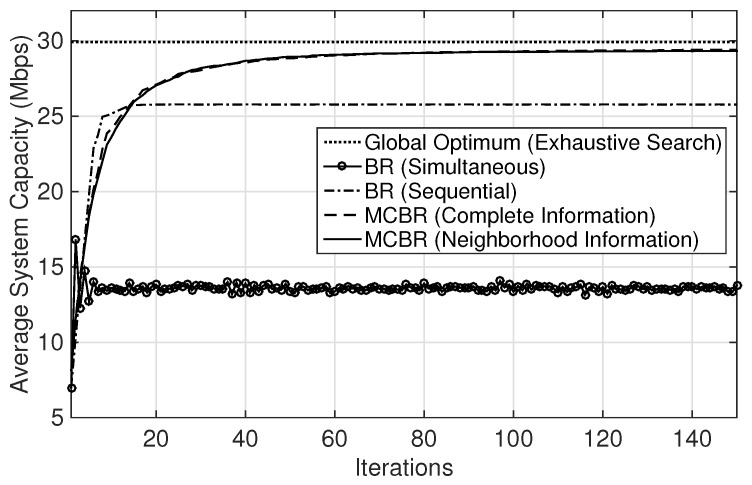
Average system capacity over 200 random deployments.

Figure 5 shows the average values of system capacity obtained for 200 random deployments of the sparse scenario. The performance of MCBR (Neighborhood Information) and MCBR (Complete Information) are close to the optimal solution (obtained from an exhaustive search). The results demonstrate the near-optimal performance of the two MCBR schemes. Note that the convergence speeds to steady states for both MCBR schemes are almost similar and exhibit convergence to maxima. The overall average performance of the system capacity is compromised in BR (Sequential) when players opportunistically try to maximize their own capacities, whereas the BR (Simultaneous) results in the worst average performance of all when all links update their strategies simultaneously.

### 5.2. Performance in Different Radio Resource Scenarios

In this subsection, we compare performance of MCBR (Neighborhood Information) with other schemes in all the three radio resource scenarios. We average the performance for 200 randomly generated deployments of each scenario, where the number of iterations is fixed to 500. Moreover, we show 95% confidence intervals in the following figures. Note that for all the schemes, the confidence intervals for the sparse scenario are too small to be visible, which is justified by the plots obtained in Figure 5.

In order to evaluate the fairness in capacities obtained by each link, we use Jain’s fairness index [38], which is expressed as,

(13) $$\frac{{\left(\sum_{i=1}^{M}C_{i}\right)}^{2}}{M\sum_{i=1}^{M}C_{i}^{2}}$$

**Figure 6:**
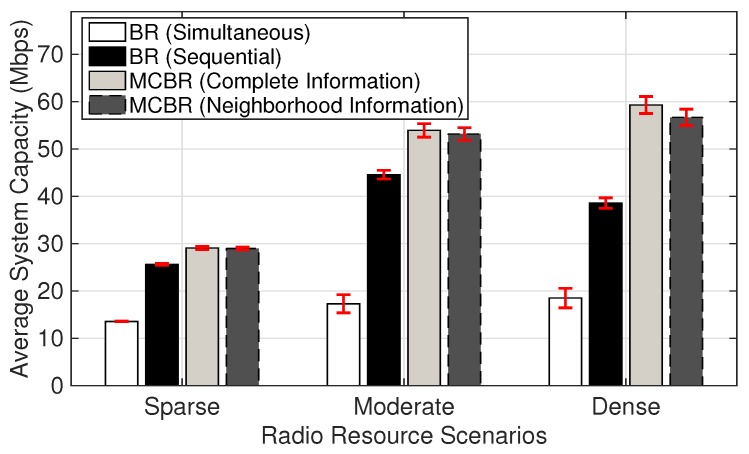
Average system capacity in different radio resource scenarios.

This fairness index is widely applied in the literature to evaluate the level of fairness achieved by resource allocation algorithms. Table 3 shows the average fairness index calculated in the three radio resource scenarios. MCBR (Neighborhood Information) is able to provide with satisfying level of fairness as compared to other schemes. BR (Simultaneous) is able to maintain the fairness above 0.9 in all the three radio resource scenarios. However, this performance result comes at an expense in efficiency as shown in Figure 6 .

**Figure 7:**
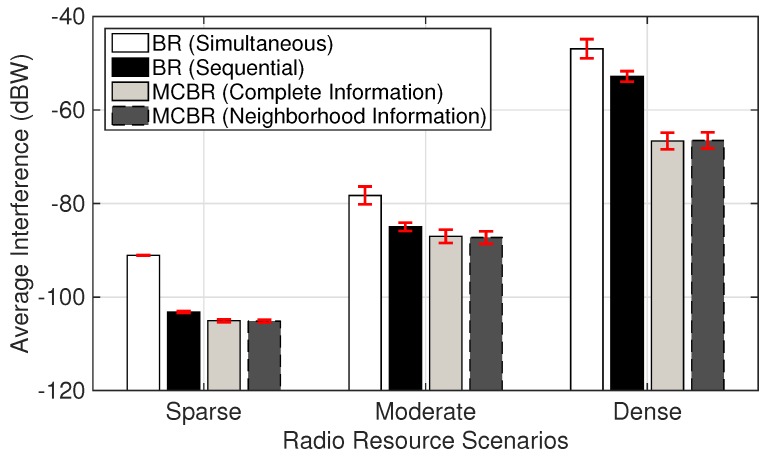
Average interference in different radio resource scenarios.

Figure 6 compares the average system capacity for the three radio resource scenarios. MCBR (Complete Information) provides better average system capacity as compared to other schemes, while considerable performance is obtained from MCBR (Neighborhood Information) in all the scenarios. With increasing number of links, the achievable average system capacity from MCBR (Complete Information) and MCBR (Neighborhood Information) increases more quickly than for the other two schemes. MCBR (Complete Information) and MCBR (Neighborhood Information) are able to provide significant performance improvement even in the dense scenarios. In addition, the accumulated interference for both MCBR schemes compared to other BR strategy schemes is considerably smaller, as seen in Figure 7.

Next in Table 4, we evaluate the reduction in average number of links from which information feedback are required for MCBR (Neighborhood Information) compared to MCBR (Complete Information) in the three radio resource scenarios. Recall that in both the MCBR schemes, each link requires feedback only from other links that are allocated to the new sensing subchannel and the subchannel allocated in the previous time-slot. Under MCBR (Complete Information), which requires information feedback from all other links in the SCN allocated to the particular sensing subchannel, the average numbers of links for information feedback are 2.65, 12.86, and 46.37 links, respectively. Since MCBR (Neighborhood Information) requires information feedback only of links originating from the neighboring SBSs, the average numbers of links for information feedback drop significantly to 0.94, 4.17, and 12.83 links, respectively, which is a 65–70% reduction in all the three radio resource scenarios. These results show that the computational complexity of MCBR (Neighborhood Information) is low, scalable, and suitable for practical implementation in different radio resource scenarios of SCNs. From these analysis, we can conclude that MCBR (Neighborhood Information) is able to provide efficient and fair subchannel allocation, even with limitations to complete information feedback in the SCN.

## 6. Conclusions

In this paper, we utilized the concept of MC to design the learning rule for the distributed subchannel allocation problem with an objective to maximize welfare of the SCNs, defined as the total system capacity. The proposed MCBR algorithm requires limited information feedback, i.e., only for the sensing subchannel from other links in the neighborhood, and is verified to be an EPG. This low and scalable computational complexity makes it suitable for practical implementation in SCNs while it attains the desirable properties of EPGs, as: (1) it ensures existence of a pure strategy NE, (2) the pure strategy NE corresponds to the maxima of the potential function, and (3) sequential updates of strategy following BR dynamics result in convergence to a pure strategy NE. Simulation results show that the proposed scheme is able to achieve more satisfactory performance than other baseline solutions in terms of convergence speed, system capacity, fairness, and interference. Moreover, it ensures an efficient performance close to that obtained with a centralized exhaustive search. From the analysis, we can conclude that efficient and fair performance is achievable for the distributed subchannel allocation problem when decision-makers are aware of their influence on others while they are trying to take an action to improve own performance.

## Figures and Tables

**Table 1:** Summary of notations. AWGN: additive white Gaussian noise; BR: best-response; SINR: signal-to-interference-plus-noise ratio.

Notation (s)	Description
N,M,K	Set of SBSs, MSs, and subchannels with cardinality N,M,and K
$M_{n}$	Number of MSs associated with SBS *n*
*B*	Spectrum bandwidth
$h_{ii}\left(k\right)$	Channel gain of transmission on link *i* on subchannel *k*
$h_{ji}\left(k\right)$	Channel gain of interference from the transmitting SBS of link *j*
	to the receiving MS of link *i* on subchannel *k*
$P_{\max}$	Maximum transmit power of each SBS
$P_{i,k}$	Transmit power of link *i* on subchannel *k*
$\gamma_{i,k}$	SINR for link *i* on subchannel *k*
$I_{i,k}$	Co-channel interference to link *i* on subchannel *k*
$\sigma^{2}$	AWGN power
$C_{i}$	Capacity of link *i*
C	Total capacity
G	Strategic form game
$S_{i}$	Set of strategies of player i∈M, $S_{i}=\left\{1,2,\ldots ,K\right\}$
$S,S_{-i}$	Cartesian product $\prod_{j\in M}S_{j}$, and $\prod_{j\in M\setminus \{i\}}S_{j}$
$s_{i}$	Strategy of player *i*, $s_{i}\in S_{i}$
$s_{-i}$	Strategy of all players except *i*, $s_{-i}\in S_{-i}$
$u_{i}$	Utility of player *i*
$BR_{i}$	BR strategy of player *i*
P	Potential function
$W_{k}\left(s\right)$	Total welfare from all links that are using subchannel *k*
$p_{i}$	Subchannel selection probability vector of link *i*
$p_{i,k}\left(t\right)$	Probability of choosing subchannel *k* for sensing at time slot *t* by link *i*
${\tilde{r}}_{i}$	Normalized utility increment of link *i*
β	Learning step-size
$r_{n}$	Circular coverage radius of SBS *n*

**Table 2:** Simulation parameters.

Parameters	Values
Carrier frequency	2 GHz
Channel bandwidth (*B*)	1.4 MHz
Number of subchannels (*K*)	{6, 9, 12, 15, 18}
SBS max. transmit power ($P_{\max}$)	20 dBm
Noise power density	−174 dBm/Hz
Neighborhood distance	30 m
Path-loss:	
Between SBS and MS link	$127+30log_{10}\left(D\right)$
Between other links	$128.1+37.6log_{10}\left(D\right)$
	where *D* is link distance in km.
Standard deviation of lognormal shadowing	10 dB (between SBS and MS link)
	8 dB (between other links)
Multipath Rayleigh fading	Exponential random variable with unit mean
Penetration loss	0 dB (between SBS and MS link)
	20 dB (between other links)

**Table 3:** Fairness comparison.

	Sparse	Moderate	Dense
BR (Simultaneous)	0.9705	0.9567	0.9483
BR (Sequential)	0.9694	0.9309	0.8647
MCBR (Complete Information)	0.9465	0.9095	0.7743
MCBR (Neighborhood Information)	0.9582	0.9197	0.8029

**Table 4:** Information feedback requirements from average number of links.

	Sparse	Moderate	Dense
MCBR (Complete Information)	2.65	12.86	46.37
MCBR (Neighborhood Information)	0.94	4.17	12.83
% reduction	64.53	67.57	72.33

## Abbreviations

The following abbreviations are used in this paper:

3GPP	Third-Generation Partnership Project
AWGN	Additive White Gaussian Noise
BR	Best Response
BS	Base Station
EPG	Exact Potential Game
MBS	Macrocell Base Station
MC	Marginal Contribution
MCBR	Marginal Contribution-Based Best Response
MS	Mobile Station
NE	Nash Equilibrium
NRT	Neighbor Relation Table
OFDMA	Orthogonal Frequency Division Multiple Access
PCI	Physical Cell Identity
PG	Potential Game
RSRP	Reference Signal Receive Power
SBS	Small Cell Base Station
SCN	Small Cell Network
SINR	Signal-to-Interference-Plus-Noise Ratio
WSN	Wireless Sensor Network
